# Viral loads correlate with upregulation of PD-L1 and worse patient prognosis in Epstein–Barr Virus-associated gastric carcinoma

**DOI:** 10.1371/journal.pone.0211358

**Published:** 2019-01-29

**Authors:** Atsuhito Nakayama, Hiroyuki Abe, Akiko Kunita, Ruri Saito, Teru Kanda, Hiroharu Yamashita, Yasuyuki Seto, Shumpei Ishikawa, Masashi Fukayama

**Affiliations:** 1 Department of Pathology, Graduate School of Medicine, the University of Tokyo, Tokyo, Japan; 2 Department of Microbiology, Tohoku Medical and Pharmaceutical University, Sendai, Japan; 3 Department of Gastrointestinal Surgery, Graduate School of Medicine, the University of Tokyo, Tokyo, Japan; University of Hong Kong, HONG KONG

## Abstract

Epstein–Barr virus (EBV)-associated gastric carcinoma (EBVaGC), one of four major gastric cancer types, consists of clonal growth of EBV-infected epithelial cells. However, the significance of viral loads in each tumor cell has not been evaluated. EBV-DNA is stably maintained in episomal form in the nucleus of each cancer cell. To estimate EBV copy number per genome (EBV-CN), qPCR of viral *EBNA1* and host *GAPDH*, standardized by Namalwa DNA (one copy/genome), was applied to the formalin-fixed paraffin embedded (FFPE) surgically resected EBVaGC specimens (n = 43) and EBVaGC cell lines (SNU-719 and NCC-24). In surgical specimens, the cancer cell ratio (CCR) was determined with image analysis, and EBV-CN was obtained by adjusting qPCR value with CCR. Fluorescent *in situ* hybridization (FISH) was also applied to the FFPE sections using the whole EBV-genome as a probe. In surgical specimens, EBV-CN obtained by qPCR/CCR was between 1.2 and 185 copies with a median of 9.9. EBV-CN of SNU-719 and NCC-24 was 42.0 and 1.1, respectively. A linear correlation was observed with qPCR/CCR data up to 20 copies/genome (40 signals/nucleus), the limit of FISH analysis. In addition, substantial variation in the number of EBV foci was observed. Based on qPCR/CCR, high EBV-CN (>10 copies) correlated with PD-L1 expression in cancer cells (*P* = 0.015), but not with other pathological indicators. Furthermore, EBVaGC with high EBV-CN showed worse disease-specific survival (*P* = 0.041). Our findings suggest that cancer cell viral loads may contribute to expression of the immune checkpoint molecule and promotion of cancer progression in EBVaGC.

## Introduction

Epstein–Barr virus (EBV)-associated gastric cancer (EBVaGC), one of the four major types of gastric cancer, consists of clonal growth of EBV-infected epithelial cells. When in situ hybridization (ISH) targeting EBV-encoded small RNA (EBER) is applied to the tissue sections of EBVaGC, all of the cancer cells show positive signals in the nuclei with extremely rare EBER-ISH-positive lymphocytes in spite of dense infiltration of lymphocytes. EBVaGC comprises 5%–10% of gastric cancer cases and has several characteristics distinct from EBV-negative gastric cancers, such as massive lymphocytic infiltration, frequent genetic mutations in *PIK3CA* and *ARID1A*, and global hypermethylation of CpG islands [1, 2].

Viral copy number in the systemic circulation is an important clinical indicator of viral diseases. The level of virus loads in the blood has been established as a useful prognostic marker for EBV-associated malignancies, such as nasopharyngeal carcinoma [3] and hematological malignancies [4, 5]. However, the viral copy number in EBV-related tumor tissue has not yet been investigated. Ryan *et al*. quantified the number of EBV genes in bulk DNA from whole-slide sections of tumor samples [6, 7]. However, the precise EBV copy number in each cancer cell has not yet been examined, because EBVaGC consists of both EBV-infected cancer cells and EBV-negative stromal cells, including many inflammatory cells.

In the present study, we used image analysis to correct the quantitative real-time PCR data of viral and host DNA by the cancer cell ratio for the estimation of EBV copy number per single genome (EBV-CN) of cancer cells. After validating the process with fluorescent *in situ* hybridization (FISH), clinicopathological significance of EBV-CN was investigated, including expression of PD-L1, which was recently demonstrated to be specific to EBVaGC among gastric cancer subtypes [8, 9].

## Materials and methods

### Tissue samples

Forty-three cases of EBVaGC from 1998 to 2012 were obtained from the tissue archive at the Department of Pathology, University of Tokyo Hospital. The selection criteria of the cases were as follows: tumor mass is clearly distinguishable from the normal mucosa, and the diameter of tumor mass is larger than 3 mm. We set this limitation for the purpose of manual macroscopic dissection of the tumor. Sections of 3-μm thickness from formalin-fixed, paraffin-embedded (FFPE) blocks were used for hematoxylin and eosin staining and immunohistochemistry, while sections of 10-μm thickness were used for DNA extraction. Clinicopathological data were collected, including the age at surgical operation, sex, location and diameter of the tumor, tumor histology based on Lauren’s classification [10], tumor depth, lymphatic and venous invasion, and metastasis to the lymph node. This study procedure including the consent procedure was approved by the Institutional Review Board of the University of Tokyo (approval number G3521). The study plan is disclosed on our website (http://pathol.umin.ac.jp/research.shtml) with a notification to patients for the opportunity to opt out of the study.

### EBER *in situ* hybridization and immunohistochemistry

EBV-encoded small RNA (EBER) *in situ* hybridization was performed with EBER peptide nucleic acid (PNA) probe/fluorescein (Y5200, Dako, Glostrup, Denmark) and the DAB Peroxidase (HRP) Substrate Kit (SK-4100, Vector Laboratories, Burlingame, CA, USA). All cases showed positive EBER signal in the tumor nuclei, which confirmed the diagnosis of EBVaGC.

Immunohistochemistry was performed with an anti-multi-cytokeratin antibody (mouse monoclonal, clone AE1/AE3, dilution 1:200; Leica Biosystems, New Castle, UK), anti-PD-L1 antibody (rabbit monoclonal, clone E1L3N, dilution 1:200; Cell Signaling Technology, Danvers, MA, USA), anti-PD-1 antibody (mouse monoclonal, clone NAT105, dilution 1:100; Abcam, Cambridge, UK), and anti-CD8 antibody (mouse monoclonal, clone 4B11, dilution 1:40; Leica Biosystems, Nussloch, Germany). The staining procedure was automated by the Ventana BenchMark XT platform and iVIEW DAB Detection Kit (F. Hoffmann-La Roche, Basel, Switzerland). Immunohistochemistry of cytokeratin was followed by digital image analysis described below. Immunohistochemical data of PD-L1, PD-1, and CD8 of all cases were provided from the previous report, which used the present case series part of the dataset [11]. Briefly, the tumor was regarded as positive for PD-L1 if more than 5% of cancer cells showed membranous staining. PD-L1 or PD-1 positive immune cell infiltration was evaluated semi-quantitatively. CD8 positive immune cell infiltration was analyzed by the digital image analysis software Tissue Studio (Definiens, Munich, Germany) dividing into high and low infiltration groups.

Data of *PD-L1* gene amplification was also provided from our previous report [11]. Briefly, fluorescent *in situ* hybridization of *PD-L1* gene was performed and amplification was analyzed. Immunohistochemical data of PD-1 and CD8 was available in 40 of 43 cases, and amplification data was available in 32 of 43 cases in the present study.

### Cell line

The Burkitt lymphoma cell line Namalwa (CRL-1432) was purchased from American Type Culture Collection (Manassas, VA, USA). The EBVaGC cell lines SNU-719 and NCC-24 were purchased from Korean cell line bank (Seoul, Korea). The EBV-negative acute monocytic leukemia cell line THP-1 (with diploid chromosomes) was obtained from Cell Resource Center for Biomedical Research, Institute of Development, Aging and Cancer, Tohoku University (Sendai, Japan). Cell lines were cultured in RPMI 1640 medium (Nacalai Tesque, Kyoto, Japan) containing 10% fetal bovine serum and 1% penicillin and streptomycin.

### DNA extraction

In all 40 EBVaGC cases, the tumor region was macroscopically dissected with a scalpel from 10-μm-thick sections, and DNA was extracted with the DNeasy Blood & Tissue Kit (QIAGEN, Hilden, Germany) according to the manufacturer’s protocol. DNA extraction from cell lines was performed with the same kit.

### Quantitative real-time PCR (qPCR)

To determine the EBV copy number, TaqMan qPCR targeting an EBV gene, *EBNA1*, and a host human gene, *GAPDH*, was designed. The fluorescent dye HEX was placed at the 5′-side of the probes. To lower the background signal, two quenchers, ZEN and IABkFQ, were conjugated to each probe (Integrated DNA Technologies, Coralville, IA, USA). Primers used in the study were as follows: *EBNA1*, forward 5′-TGG CCC AGA TGG TGA GCC-3′, reverse 5′-CCT GCC TCC ATC ACC CTG A-3′, probe 5′/HEX/CGG GAG CGA/ZEN/TAG AGC AGG GC/IABkFQ/3′; and *GAPDH* forward 5′-TAG AGG GGT GAT GTG GGG AG-3′, reverse 5′-AGT GAT GGC ATG GAC TGT GG-3′, probe 5′/HEX/AAG GAG TGA/ZEN/GGC CCT GCA GC/IABkFQ/3′. Primers and probe for *EBNA1* were designed to avoid mutation sites of Namalwa. We also confirmed that the sequences did not include the previously reported mutations of EBV in 9 EBVaGC cases and two EBVaGC cell lines (SNU-719 and YCCEL1) [12, 13]. qPCR was performed using the Applied Biosystems 7300 Real Time PCR System (Applied Biosystems, Foster City, CA, USA) with 2× TaqMan Gene Expression Master Mix (Thermo Fisher Scientific, Waltham, MA, USA), a mix of primers and a probe for *EBNA1* or *GAPDH*, and 30 ng of template DNA. Since both primers were labeled with HEX, assays for each gene were performed in separate wells. Thermal cycling profiles were 2 min at 50°C, 10 min at 95°C, and 40 cycles of 15 seconds at 95°C and 1 min at 60°C. For absolute quantification, genomic DNA from Namalwa was used as a standard sample. Namalwa contains two copies of EBV tandemly inserted into chromosome 1 [14]. Therefore, the *EBNA1*/*GAPDH* ratio, which is defined as the ratio of *EBNA1* and *GAPDH* gene copy number, is assumed to be 1 in Namalwa cells. Four different amounts of Namalwa DNA (100 ng, 50 ng, 10 ng, and 2 ng) were tested to draw standard quantification curves. Sample DNA was tested in triplicate, and the mean *EBNA1*/*GAPDH* ratio in the bulk DNA was estimated from the standard curve. The amplified products were electrophoresed by the MultiNA Microchip Electrophoresis System (Shimadzu, Kyoto, Japan), and bands of the expected length were confirmed.

In addition, to determine the lower limit of detection of our analyses, we also performed qPCR using mixed DNA samples from Namalwa and THP-1 cells in various proportions (1, 1:2, 1:10, 1:100, and 1:1000).

### Digital image analysis and estimation of EBV copy number per genome (EBV-CN)

To determine the cancer cell ratio, the number of nuclei of cancer and stromal cells in the macroscopically dissected areas was measured by digital image analysis. The whole-slide images of cytokeratin-imunostained slides of EBVaGC were captured by NanoZoomer 2.0-HT (Hamamatsu Photonics, Hamamatsu, Japan). The digital images were analyzed using the tissue image analysis software Tissue Studio. The immunostained cancer area was distinguished from the stroma, and the number of nuclei was automatically calculated in the cancer and stromal areas, providing the ratio of cancer cell nuclei to the whole cell nuclei (CCR).

Viral copy number per genome (EBV-CN) was calculated by dividing the *EBNA1*/*GAPDH* ratio by the CCR. EBV-CN refers to the number of *EBNA1* copies per single *GAPDH* copy in the cancer cell nucleus, presuming the same copy number of *GAPDH* in the cancer and stromal cell nucleus.

### FISH analysis of the EBV genome

A 174-kbp FISH probe was designed complementary to the template bacmid of the whole EBV genome [15], and Texas Red was conjugated to the probe (GSP Lab, Kobe, Japan). A FITC-conjugated control probe that binds near the centromere of chromosome 17 (CEP17) was obtained from GSP Lab. FISH was performed in Namalwa cells and 9 EBVaGC cases according to the manufacturer’s instructions. In brief, a 4-μm-thick section was deparaffinized in xylene and hydrophilized in 100% ethanol. The slides were then immersed in the pretreatment solution (GSP Lab, Kobe, Japan) at 100°C for 30 min. After proteolytic treatment at 37°C for 15 min, EBV and CEP17 probes were added. The slides were heated at 75°C for 5 min, incubated at 37°C overnight, and washed in 2× SSC solution. The nuclei were counterstained with DAPI. The slides were observed under Leica DM6000B microscopy with Leica Application Suite X software (Leica Microsystems). We selected several areas in each case, and a total of 19 pictures (1000× magnification) were consecutively obtained along the z axis over the thickness of 9 μm at an interval of 0.5 μm. In each area, about 40 tumor nuclei containing both EBV and CEP17 signal were selected and the number of EBV signals per tumor nucleus was manually counted.

For comparison between EBV-CN and the number of FISH signals, a four-parameter logistic model, also known as the Hill equation, was fitted to 10 data points including (0, 0) and the averaged values from 9 cases. GraphPad Prism 6 estimated the optimal coefficients of the Hill equation.

FISH of cell lines were also performed by preparing cell blocks. In brief, cells were collected and fixed in 4% paraformaldehyde, then put into agarose gel and embedded in paraffin blocks. Cell blocks were processed in the same way as surgical specimens, then EBV and CEP17 foci were manually counted.

### Statistical analyses

JMP Pro 11 (SAS, Buckinghamshire, UK) and GraphPad Prism 6 (GraphPad Software, La Jolla, CA, USA) were used for statistical analyses and graphical depiction of the data. Correlations between high or low EBV-CN and the clinicopathological factors were examined by two-sided Fisher’s exact test. The relationships between EBV-CN and PD-L1 expression were analyzed by two-sided Fisher’s exact test and Mann-Whitney *U* test.

Overall survival (OS) was defined as the time from surgical resection to death of any cause or the last follow-up. Disease-specific survival (DSS) was defined as the time from surgical resection to death solely due to EBVaGC or the last follow-up. The survival time was compared by generalized Wilcoxon’s test. Multivariate analysis could not be performed in the current study due to the absence of events in cases with stage pT1 or no lymph node metastasis.

## Results

### EBV viral copy number per tumor genome (EBV-CN)

First, we performed qPCR on DNA extracted from Namalwa cells (two EBV copies per cell) and THP-1 cells (with no EBV infection and diploid chromosomes) in various proportions. We were able to detect the EBV genome in mixed DNA samples from Namalwa and THP-1 cells even when the ratio of Namalwa:THP-1 cell DNA was 1:1000 (S1 Fig). These results assured high sensitivity of the qPCR method.

**Fig 1:**
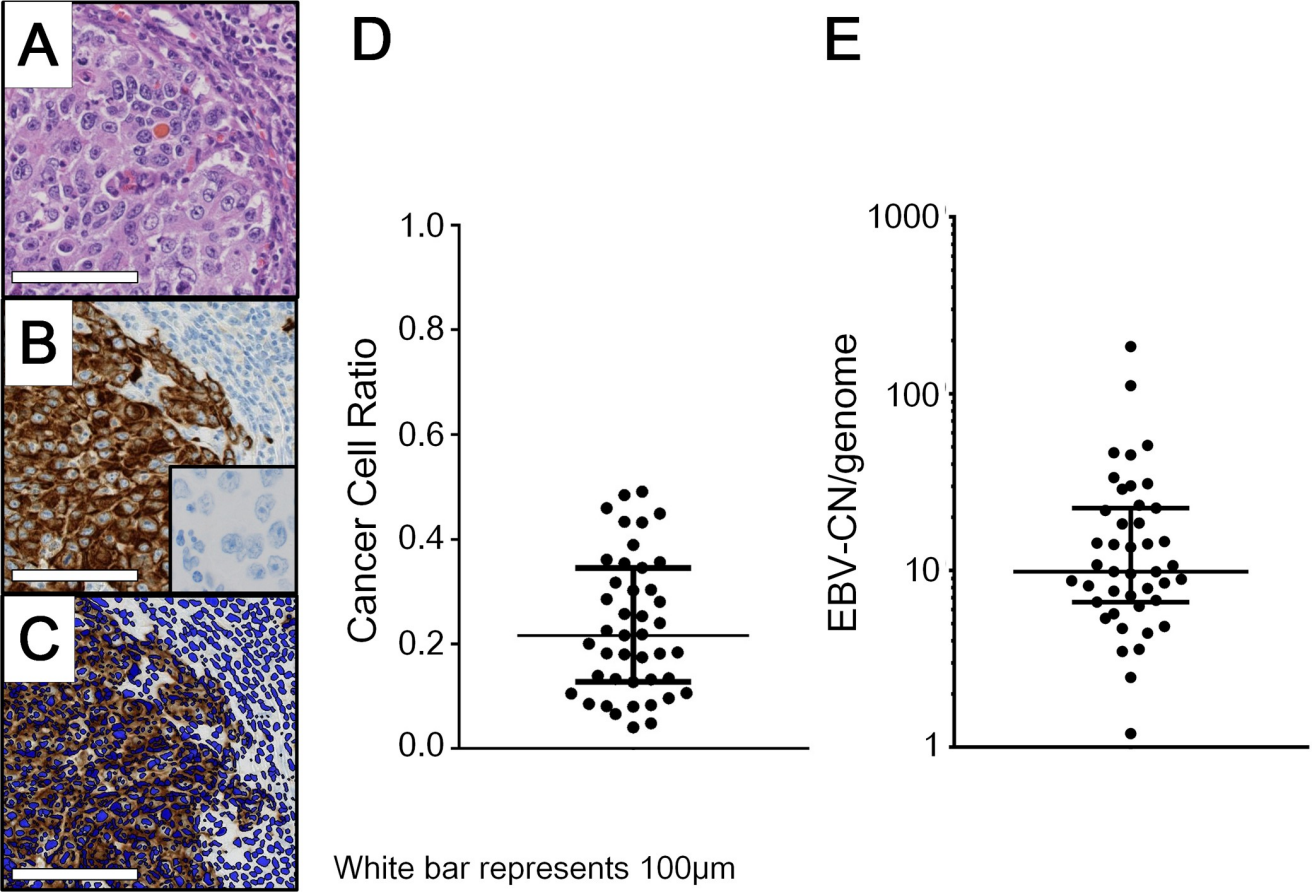
Copy number of EBV-DNA in EBV-associated gastric carcinoma. Estimation of cancer cell ratio (CCR) was calculated for the adjustment of qPCR data. An EBV-associated gastric carcinoma (EBVaGC) case (A, hematoxylin and eosin staining) was immunostained with the monoclonal antibody AE1/AE3 (B). Using immunohistochemistry images, all the nuclei (blue) were automatically counted by the Tissue Studio software in the whole and cytokeratin immunostained regions (C). The CCR in EBVaGC cases (n = 43) distributes 4.1%–49% with a median of 22% (D). EBV-CN was obtained from qPCR data divided by the CCR (qPCR/CCR). Copy numbers of EBV-DNA per genome (EBV-CN) in EBVaGC cases (n = 43) are in the range between 1.2 and 185 with a median of 9.9 (E). A–C: Scale bar represents 100 μm. B: Inset is negative control, in which staining was performed without primary antibody. D, E: The bars represent the median and quartiles.

Using the conditions of qPCR presented in this study, the *EBNA1*/*GAPDH* ratio in EBVaGC cancer tissues demonstrated a median of 1.9 and range of 0.2–44. The ratio of cancer cell nuclei to whole nuclei (CCR) was obtained by analyzing the cytokeratin-immunostained slides with the image analyzer. CCR demonstrated a median of 22% and range of 4.1%–49% (Fig 1A–1D). The EBV-CN was calculated by dividing the *EBNA1*/*GAPDH* ratio by CCR, resulting in a median of 9.9 and range of 1.2 and 185 (Fig 1E). Cell lines (SNU-719 and NCC-24) were also analyzed by qPCR. As for the cell blocks of the cell lines, adjustment of digital image analyses was unnecessary. EBV-CN was 42.0 in SNU-719 and 1.1 in NCC-24.

### Validation of EBV-CN by EBV-FISH

**Fig 2:**
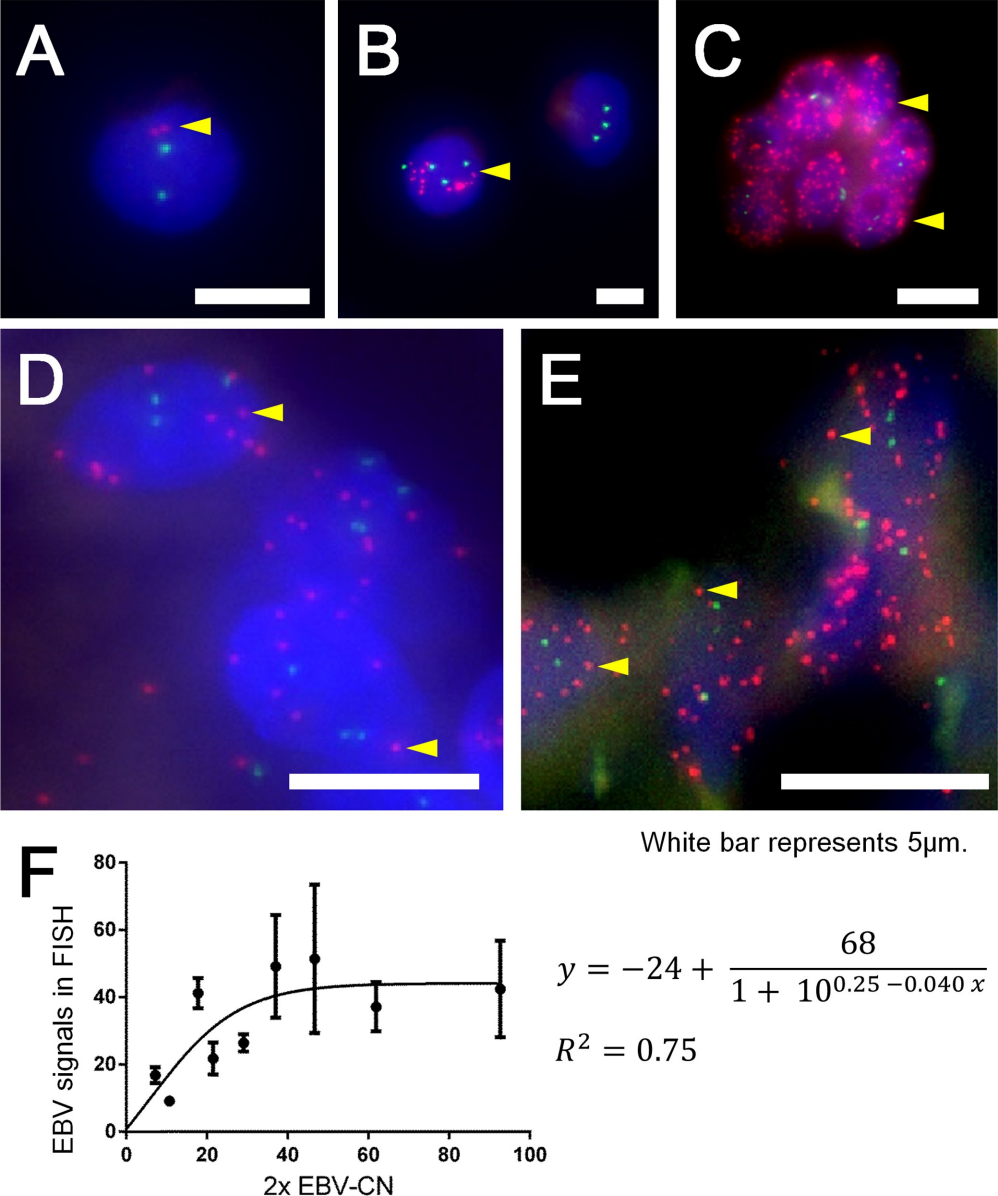
Fluorescent *in situ* hybridization of EBV and its correlation with qPCR/CCR data in EBV-associated gastric carcinoma. Fluorescent *in situ* hybridization (FISH) of EBV-DNA was performed with a specific DNA probe against the full-length EBV genome. Each red signal represents a single copy of the EBV genome. Each nucleus of EBV-infected cell line Namalwa contains one or two signals in each cell (A). Each nucleus of NCC-24 contains a lot of red signals in some cells, while no red signal in others, resulting in the average 3.9/nucleus (B). SNU-719 shows a lot of red signals in each nucleus, 34 on average. There are 7 nuclei in the figure (C). In two EBV-associated gastric carcinoma (EBVaGC) cases, the one shows 9 signals per nucleus (D) and the other 26 signals per nucleus (E). In both figures, several nuclei are aggregated. The graph presents correlation of FISH analysis with qPCR/CCR data (F). The y-axis represents the average number of EBV per nucleus by FISH. The x-axis represents the EBV copy number per nucleus, which was twice the corresponding value of the EBV-CN, assuming no amplification or deletion of *GAPDH* in the cancer cell nucleus. Dots and bars represent the mean and standard deviation, respectively. For the cases in which 2 × EBV-CN is less than 40, the number of EBV signals in FISH is proportional to 2 × EBV-CN; it gradually comes close to a plateau of 44 when 2 × EBV-CN exceeds 40 (*R*^2^ = 0.75 on the Hill curve fitting). A-E: Scale bar is 5 μm. Yellow arrowheads point to a single red signal of the EBV genome in each figure.

For validation of EBV-CN, the number of EBV signals in the tumor nucleus was directly counted in FISH-stained slides. The Texas Red-conjugated probe against the whole-length EBV genome spotted EBV at a single-copy resolution. In Namalwa cells, one or two EBV signals were observed (Fig 2A), as previously reported [14]. In gastric cancer cell lines, the number of foci per nucleus was 34±16 in SNU-719 and 3.9±7.3 in NCC-24 (Fig 2B and 2C). EBVaGC clinical specimens contained various numbers of EBV signals. Two representative cases in Fig 2D and 2E showed cases with 9 and 26 EBV signals, respectively. The numbers of EBV signals were counted in 36–50 nuclei in each region of interest (ROI). The average number represented the EBV signals/nucleus in each ROI. Count data of EBV foci is available in S1 File.

On the assumption that a single tumor nucleus contains two copies of *GAPDH*, 2 × EBV-CN was compared with the number of EBV signals/nucleus. Distribution of the data points appeared to show proportional relationships with a plateau at a critical point around (40, 40). To justify this observation, a non-linear model based on the Hill equation was fitted (Fig 2F). In the optimized model with *R*^2^ value of 0.75, the EBV signals/nucleus increased until 2 × EBV-CN exceeded around 40, and it became asymptotic to a line, *y* = 44.

### EBV-CN correlated with PD-L1 expression but not with clinicopathological features

**Fig 3:**
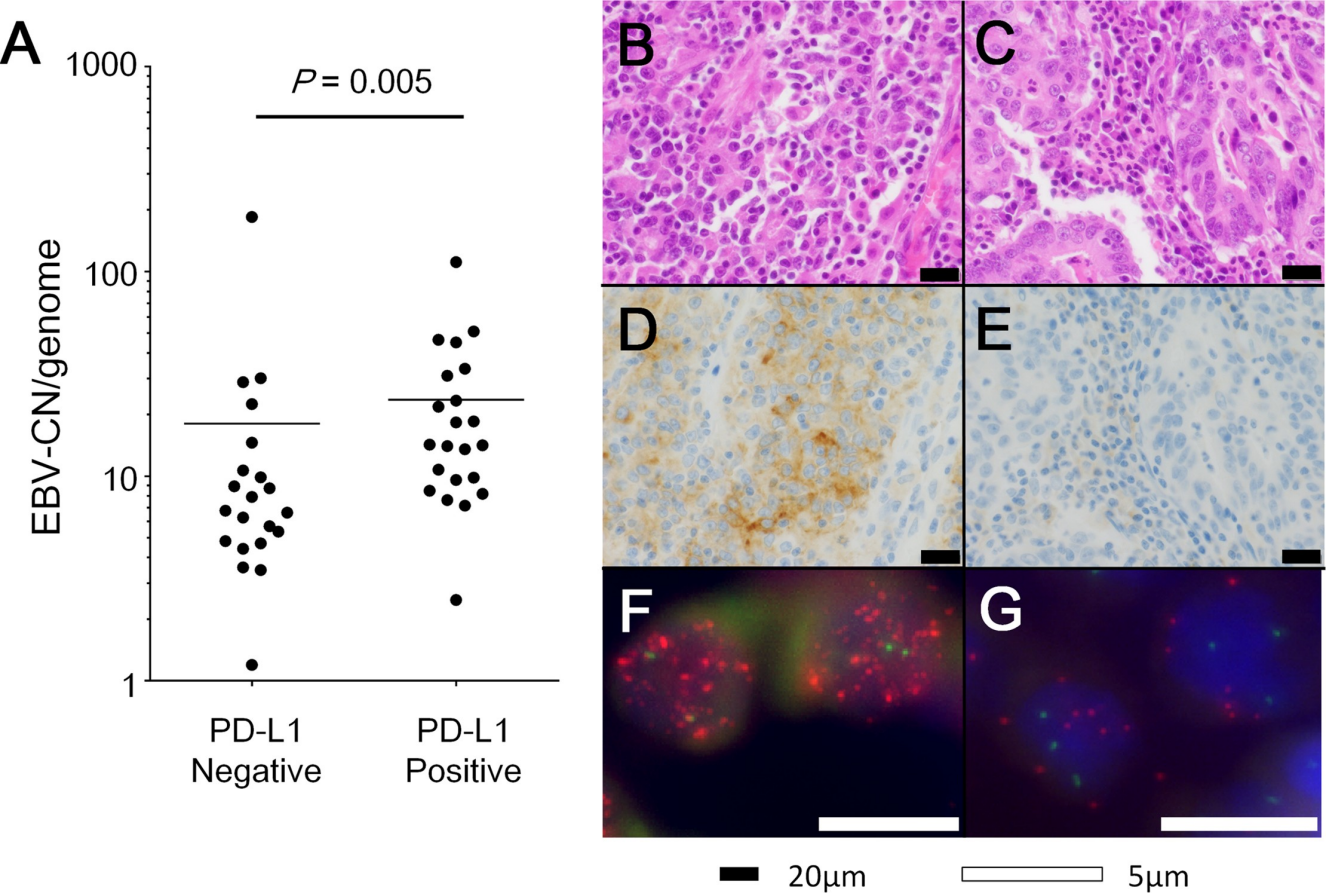
Association of PD-L1 expression with EBV-CN or EBV-FISH. Comparison of EBV-CN between PD-L1-positive and -negative tumors revealed significantly higher EBV-CN in PD-L1-positive tumors (*P* = 0.005, Mann-Whitney *U* test) (A). Representative images of hematoxylin and eosin staining, PD-L1 immunohistochemistry, and EBV-FISH of an EBVaGC case with 41 EBV signals per nucleus (B, D, and F, respectively) and an EBVaGC case with 9 EBV signals per nucleus (C, E, and G, respectively). B, C: Hematoxylin and eosin staining; D, E: PD-L1 immunohistochemistry; F, G: EBV-FISH.

The total 43 EBVaGC cases were divided into the low EBV-CN group (n = 22) and the high EBV-CN group (n = 21) at a threshold of the median (9.9). There was no clinicopathological factor significantly associated with EBV-CN. However, immunohistochemical analysis revealed that high EBV-CN significantly correlated with positive expression of PD-L1 in cancer cells (*P* = 0.015), while no correlation was observed with expression of PD-L1, CD8 and PD-1 in stromal immune cells or amplification of *PD-L1* gene in cancer cells (Table 1). Comparison of EBV-CN between PD-L1-positive and -negative tumors revealed that EBV-CN was significantly higher in PD-L1-positive tumors (*P* = 0.005, Mann-Whitney *U* test) (Fig 3A). Representative images of PD-L1 immunohistochemistry and EBV-FISH are presented in parallel for an EBVaGC case with 40 EBV-CN (Fig 3B, 3D and 3F) and 9 EBV-CN (Fig 3C, 3E and 3G), respectively.

**Table 1:** EBV-CN and clinicopathological characteristics of EBVaGC.

Variable		Low EBV-CN group (n = 22)	High EBV-CN group (n = 21)	*P* value
Age at surgery (yr)	≤65	12	8	0.36
	>65	10	13	
Sex	Male	16	15	1
	Female	6	6	
Tumor Location	Upper	10	12	0.55
	Middle or Lower	12	9	
Diameter (mm)	≤50	12	10	0.76
	>50	10	11	
Lauren’s classification	Diffuse	6	8	0.53
	Intestinal	16	13	
Depth of invasion	Early	8	8	1
	Advanced	14	13	
Lymphatic invasion	Absent	15	10	0.22
	Present	7	11	
Venous invasion	Absent	6	4	0.72
	Present	16	17	
Lymph node metastasis	Absent	15	11	0.36
	Present	7	10	
PD-L1 in tumor cells	Negative	15	6	0.015*
	Positive	7	15	
PD-L1 in immune cells	Low	10	5	0.20
	High	12	16	
PD-1 in immune cells	Low	7	4	0.29
	High	12	17	
CD8 in immune cells	Low	10	6	0.20
	High	9	15	
*PD-L1* amplification**	Absent	2	3	
	Present	12	15	

EBV-CN, EBV viral copy number per tumor genome; EBVaGC, EBV-associated gastric carcinoma.

*P* values were calculated by two-sided Fisher’s exact test.

*P < 0.05.

**Amplification data was available only in 32 cases, which were studied in our previous report [11].

### EBV-CN and patient prognosis

**Fig 4:**
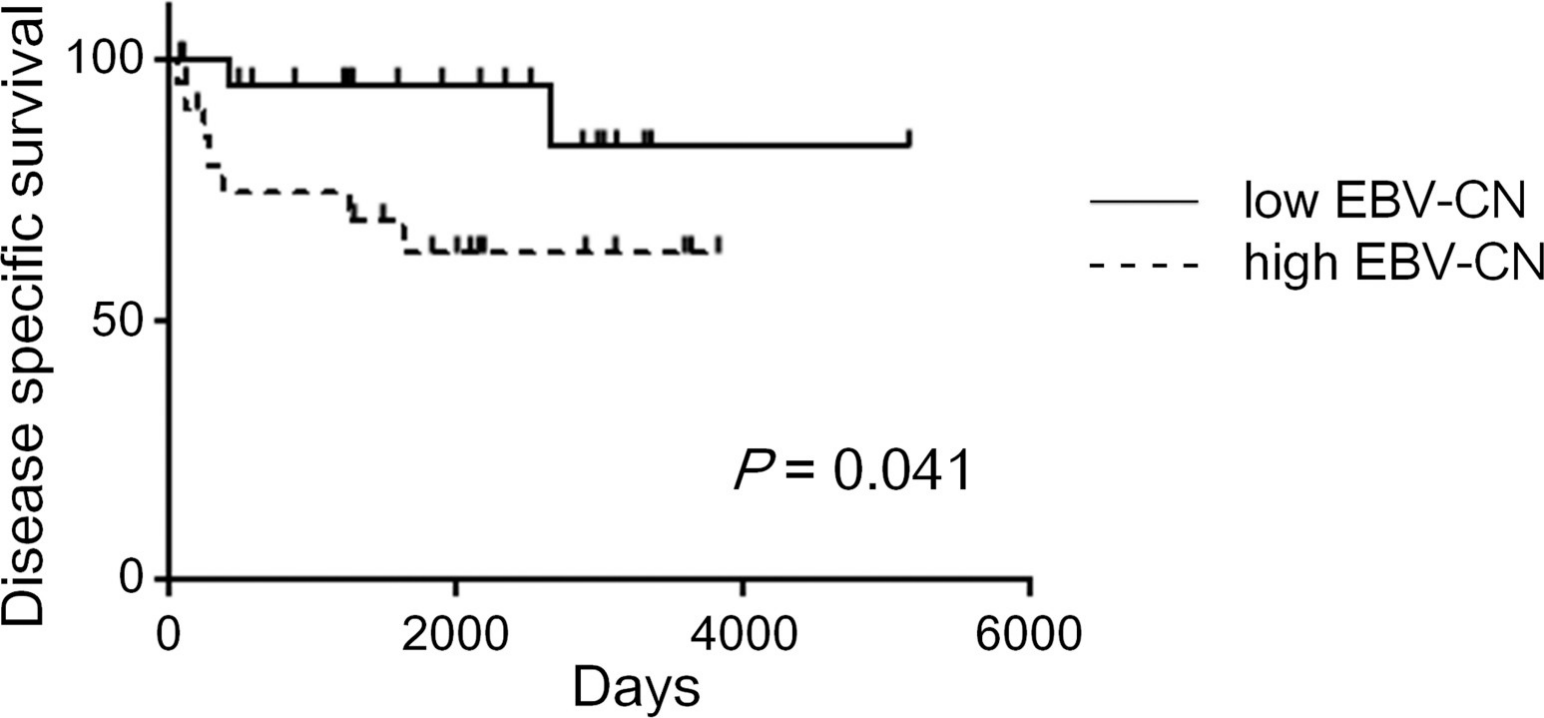
Prognostic significance of viral burden in cancer cells in EBV-associated gastric carcinoma. Disease-specific survival of patients with EBVaGC comparing low and high EBV-CN groups. The median of EBV-CN was used as a threshold. Note the shorter survival in patients with high EBV-CN (*P* = 0.041).

High EBV-CN was significantly associated with poorer prognosis of the patients in DSS (*P* = 0.041). High EBV-CN also showed tendency of worse prognosis in OS, although not statistically significant (*P* = 0.069). (Fig 4 and S2 Fig). Tumor size, invasion depth, lymphatic invasion, and lymph node metastasis were also correlated with DSS of the patients. However, because of the low number of events, especially absence of events in cases with pT1 tumor or without lymph node metastasis, multivariate analyses of DSS could not be performed.

## Discussion

EBVaGC consists of clonal growth of stomach epithelial cells that are infected with EBV. EBV-DNA is present in the episomal form in each cancer cell nucleus and the EBV-CN is stably maintained along the chromatin partitioning. Many studies have investigated the role of viral latent gene products in EBVaGC, but few studies have focused on the significance of viral load in cancer cells. In the present study, EBV-CN was associated with a worse survival rate of patients with EBVaGC. Since there was no association of EBV-CN with clinicopathological features, viral loads themselves might affect the behavior of EBV-infected cancer cells.

It is of interest to note that high EBV-CN was correlated with PD-L1 expression in cancer cells. According to our previous study, one-third of EBVaGC cases expressed PD-L1 in cancer cells, which was significantly frequent compared to other subtypes of gastric cancer, such as MLH1-negative, diffuse and intestinal subtypes [11]. Gene amplification of PD-L1 was observed only in a subset of PD-L1 expressing cells [11]. A previous study in EBVaGC cell lines also reported that both SNU-719 and NCC-24 cells expressed PD-L1, and the expression was upregulated by interferon-γ. Under interferon-γ treatment, PD-L1 expression was higher in SNU-719 cells compared with NCC-24 cells, although the difference was small [16]. The mechanisms of PD-L1 expression in EBVaGC thus remains to be elucidated. Viral genomes in the episomal form might directly induce expression of PD-L1 or indirectly induce PD-L1 expression through increased expression of latent genes, such as EBNA1, LMP-2A, EBERs or EBV microRNAs. Our preliminary analysis showed that the expression level of viral transcripts, such as LMP-2A and microRNAs (BART4 and 7), was higher in SNU-719 cells (high EBV-CN) than NCC-24 cells (low EBV-CN) (S1 Table). A recent study revealed that LMP-1 induces PD-L1 expression through promoter and enhancer activity in EBV-transformed lymphoblastoid cell lines [17]. EBNA2 induces expression of PD-L1 in lymphoma cells through downregulation of miR-34a [18], which targets PD-L1 [19]. These studies demonstrate that EBV uses PD-L1 to evade immunosurveillance, which might be also the case in EBVaGC, although both LMP-1 and EBNA2 are not expressed in EBVaGC. In melanoma, CD8^+^ T cells within the tumor microenvironment release interferon-γ, which stimulates melanoma cells to present PD-L1 [20]. A similar molecular mechanism might be occurring in EBVaGC. Most infiltrating T cells express CD8 in EBVaGC [21] and the interferon-γ signaling pathway is highly activated in transcriptomic analysis of EBVaGC cancer tissues [22]. Viral loads might affect the microenvironment through release of viral gene products in a form of exosome [23].

Targeting PD-1/PD-L1 in cancer therapy has a risk of immune-related adverse events [24]. Since EBV infection itself is thought to be associated with some autoimmune diseases such as multiple sclerosis [25], PD-L1-targeted therapy of EBVaGC, which might induce EBV reactivation and cellular lysis, should be performed with special caution for immune-related adverse events.

The current results may provide a rationale for targeting the virus for treatment of this virus-driven gastric cancer. Chemotherapeutic agents for gastric cancer, including cisplatin and 5-FU, are known to induce the lytic cycle of EBV and render EBVaGC cells susceptible to ganciclovir, an inhibitor of viral DNA polymerase [26]. However, it is not known whether induction of production of viral particles increases the copy number of EBV in residual cancer cells by re-infection. Monitoring of EBV-CN over the chemotherapeutic course might provide useful insights into future treatment.

In the present study, we evaluated viral copy number by two methods, qPCR corrected by cancer cell ratio (qPCR/CCR) and FISH using a probe against the entire EBV genome. The plateau was observed in the FISH analysis, suggesting some technical limitations, such as overlapping of the red fluorescent signals within the nucleus and breakage of the tumor nuclei from the sectioning. Nevertheless, the linear correlation up to 20 copies per genome (40 signals per nucleus) supports the feasibility of the current qPCR/CCR method for estimation of viral loads in EBVaGC. The fact might be due to low frequency of chromosomal abnormalities in EBVaGC [1]. The signal number of EBV by FISH analysis, even below 40 signals, showed some variation in the present study. Cancer cell lines also showed considerable variation in the number of EBV foci among cells. It might be also caused by the technical problems inherent to the use of tissue sections rather than isolated cells. However, a host or viral mechanism maintaining EBV copy number might be deregulated in some cancer cells. Nanbo *et al*. observed distributions of EBV-derived plasmids in single live cells throughout the cell cycle and found that defect in plasmid synthesis and partitioning results in variation of plasmid number in clonal populations of cells [27]. Chen *et al*. modified the EBV genome by disrupting one binding site of CTCF, a chromatin regulatory factor of the host [28]. The lymphocytes infected with the mutant EBV harbored a larger number of the viral genome compared with wild-type EBV. These experiments suggest a potential change in viral copy number secondary to certain genetic mutations.

In conclusion, the present study successfully quantified EBV-CN in EBVaGC. Viral load in cancer cells may contribute to the expression of the immune checkpoint molecule and promotion of cancer progression in EBVaGC. The current results provide rationale for targeting EBV for treatment of this virus-driven gastric cancer.

## Supporting information

S1 Fig EBNA1 detection in mixed DNA samples from Namalwa and THP-1 cells in various proportions. DNA extracted from Namalwa cells (two EBV copies per cell) and THP-1 cells (no EBV infection) was mixed in various proportions (1, 1:2, 1:10, 1:100, and 1:1000), and qPCR was performed. Even in samples with low Namalwa cell DNA concentration (1:1000), we were able to successfully detect EBNA1 DNA.
(TIF)

S2 Fig Overall survival and viral burden in cancer cells in EBV-associated gastric carcinoma. The cases of EBV-associated gastric carcinoma (EBVaGC) were classified into two groups, low and high EBV-copy number per genome (EBV-CN) groups, with the median as a threshold. Overall survival of the patients with EBVaGC was plotted. There was a tendency of worse overall survival in patients with high EBV-CN, although the difference was not statistically significant (*P* = 0.069).
(TIF)

S1 Table Expression level of viral transcripts in SNU-719 and NCC-24. (DOCX)

S1 File Number of EBV foci in FISH analyses. (XLSX)

## References

[1] The Cancer Genome Atlas Research Network. Comprehensive molecular characterization of gastric adenocarcinoma. Nature 2014;513:202–9. 10.1038/nature13480 25079317 PMC4170219

[2] Abe H, Kaneda A, Fukayama M. Epstein-Barr Virus-associated gastric carcinoma: use of host cell machineries and somatic gene mutations. Pathobiology 2015;82:212–23. 10.1159/000434683 26337667

[3] Lin JC, Wang WY, Chen KY, Wei YH, Liang WM, Jan JS et al Quantification of plasma Epstein-Barr virus DNA in patients with advanced nasopharyngeal carcinoma. N Engl J Med 2004;350:2461–70. 10.1056/NEJMoa032260 15190138

[4] Grywalska E, Rolinski J, Pasiarski M, Korona-Glowniak I, Maj M, Surdacka A, et al High viral loads of Epstein-Barr Virus DNA in peripheral blood of patients with chronic lymphocytic leukemia associated with unfavorable prognosis. PLoS ONE 2015;10:e0140178 10.1371/journal.pone.0140178 26460692 PMC4603951

[5] Hohaus S, Santangelo R, Giachelia M, Vannata B, Massini G, Cuccaro A, et al The viral load of Epstein-Barr Virus (EBV) DNA in peripheral blood predicts for biological and clinical characteristics in Hodgkin lymphoma. Clin Cancer Res 2011;17:2885–92. 10.1158/1078-0432.CCR-10-3327 21478335

[6] Ryan JL, Fan HX, Glaser SL, Schichman SA, Raab-Traub N, Gulley ML. Epstein-Barr virus quantitation by real-time PCR targeting multiple gene segments: a novel approach to screen for the virus in paraffin-embedded tissue and plasma. J Mol Diagn 2004;6:378–85. 10.1016/S1525-1578(10)60535-1 15507678 PMC1867486

[7] Ryan JL, Morgan DR, Dominguez RL, Thorne LB, Elmore SH, Mino-Kenudson M, et al High levels of Epstein-Barr virus DNA in latently infected gastric adenocarcinoma. Lab Invest 2009;89:80–90. 10.1038/labinvest.2008.103 19002111 PMC2612099

[8] Derks S, Liao XY, Chiaravalli AM, Xu XS, Camargo MC, Solcia E, et al Abundant PD-L1 expression in Epstein-Barr Virus-infected gastric cancers. Oncotarget 2016;7:32925–32. 10.18632/oncotarget.9076 27147580 PMC5078063

[9] Li Z, Lai Y, Sun L, Zhang X, Liu R, Feng G, et al PD-L1 expression is associated with massive lymphocyte infiltration and histology in gastric cancer. Hum Pathol 2016;55:182–9. 10.1016/j.humpath.2016.05.012 27260946

[10] Lauren P. The two histological main types of gastric carcinoma: diffuse and so-called intestinal-type carcinoma. An attempt at a histo-clinical classification. Acta Pathol Microbiol Scand 1965;64:31–49 14320675 10.1111/apm.1965.64.1.31

[11] Saito R, Abe H, Kunita A, Yamashita H, Seto Y, Fukayama M. Overexpression and gene amplification of PD-L1 in cancer cells and PD-L1+ immune cells in Epstein-Barr virus-associated gastric cancer: the prognostic implications. Mod Pathol 2017;30:427–439. 10.1038/modpathol.2016.202 27934877

[12] Kanda T, Furuse Y, Oshitani H, Kiyono T. Highly Efficient CRISPR/Cas9-Mediated Cloning and Functional Characterization of Gastric Cancer-Derived Epstein-Barr Virus Strains. J Virol 2016;90:4383–93. 10.1128/JVI.00060-16 26889033 PMC4836357

[13] Liu Y, Yang W, Pan Y, Ji J, Lu Z, Ke Y. Genome-wide analysis of Epstein-Barr virus (EBV) isolated from EBV-associated gastric carcinoma (EBVaGC). Oncotarget 2016;7:4903–14. 10.18632/oncotarget.6751 26716899 PMC4826252

[14] Lawrence JB, Villnave CA, Singer RH. Sensitive, high-resolution chromatin and chromosome mapping in situ: Presence and orientation of 2 closely integrated copies of EBV in a lymphoma line. Cell 1988;52:51–61. 2830981 10.1016/0092-8674(88)90530-2

[15] Kanda T, Kamiya M, Maruo S, Iwakiri D, Takada K. Symmetrical localization of extrachromosomally replicating viral genomes on sister chromatids. J Cell Sci 2007;120:1529–39. 10.1242/jcs.03434 17405814

[16] Sasaki S, Nishikawa J, Sakai K, Iizasa H, Yoshiyama H, Yanagihara M et al EBV-associated gastric cancer evades T-cell immunity by PD-1/PD-L1 interactions. Gastric Cancer 2018 10.1007/s10120-018-0880-4 [Epub ahead of print] 30264329

[17] Green MR, Rodig S, Juszczynski P, Ouyang J, Sinha P, O'Donnell E et al Constitutive AP-1 activity and EBV infection induce PD-L1 in Hodgkin lymphomas and posttransplant lymphoproliferative disorders: implications for targeted therapy. Clin Cancer Res. 2012;18:1611–8. 10.1158/1078-0432.CCR-11-1942 22271878 PMC3321508

[18] Anastasiadou E, Stroopinsky D, Alimperti S, Jiao AL, Pyzer AR, Cippitelli C et al Epstein-Barr virus-encoded EBNA2 alters immune checkpoint PD-L1 expression by downregulating miR-34a in B-cell lymphomas. Leukemia. 2018 10.1038/s41375-018-0178-x [Epub ahead of print] 29946193 PMC6327052

[19] Pyzer AR, Stroopinsky D, Rosenblatt J, Anastasiadou E, Rajabi H, Washington A et al MUC1 inhibition leads to decrease in PD-L1 levels via upregulation of miRNAs. Leukemia. 2017;31:2780–2790. 10.1038/leu.2017.163 28555079 PMC5791150

[20] Spranger S, Spaapen RM, Zha Y, Williams J, Meng Y, Ha TT et al Up-regulation of PD-L1, IDO, and Tregs in the melanoma tumor microenvironment is driven by CD8+ T cells. Sci Transl Med 2013;5:200ra116 10.1126/scitranslmed.3006504 23986400 PMC4136707

[21] Kuzushima K, Nakamura S, Nakamura T, Yamamura Y, Yokoyama N, Fujita M et al Increased frequency of antigen-specific CD8+ cytotoxic T lymphocytes infiltrating an Epstein-Barr virus-associated gastric carcinoma. J Clinical Invest 1999;104:163–71. 10411545 10.1172/JCI6062 PMC408473

[22] Strong MJ, Xu GR, Coco J, Baribault C, Vinay DS, Lacey MR, et al Differences in gastric carcinoma microenvironment stratify according to EBV infection intensity: implications for possible immune adjuvant therapy. PLoS Pathog 2013;9:e1003341 10.1371/journal.ppat.1003341 23671415 PMC3649992

[23] Pegtel DM, Cosmopoulos K, Thorley-Lawson DA, van Eijndhoven MA, Hopmans ES, Lindenberg JL, et al Functional delivery of viral miRNAs via exosomes. Proc Natl Acad Sci U S A. 2010;107:6328–33. 10.1073/pnas.0914843107 20304794 PMC2851954

[24] Khoja L, Day D, Wei-Wu Chen T, Siu LL, Hansen AR. Tumour- and class-specific patterns of immune-related adverse events of immune checkpoint inhibitors: a systematic review. Ann Oncol. 2017;28:2377–2385. 10.1093/annonc/mdx286 28945858

[25] Veroni C, Marnetto F, Granieri L, Bertolotto A, Ballerini C, Repice AM et al Immune and Epstein-Barr virus gene expression in cerebrospinal fluid and peripheral blood mononuclear cells from patients with relapsing-remitting multiple sclerosis. J Neuroinflammation. 2015;12:132–149. 10.1186/s12974-015-0353-1 26169064 PMC4501166

[26] Feng WH, Israel B, Raab-Traub N, Busson P, Kenney SC. Chemotherapy induces lytic EBV replication and confers ganciclovir susceptibility to EBV-positive epithelial cell tumors. Cancer Res 2002;62:1920–6. 11912175

[27] Nanbo A, Sugden A, Sugden B. The coupling of synthesis and partitioning of EBV's plasmid replicon is revealed in live cells. EMBO J. 2007;26:4252–62. 10.1038/sj.emboj.7601853 17853891 PMC2000340

[28] Chen HS, Martin KA, Lu F, Lupey LN, Mueller JM, Lieberman PM et al Epigenetic deregulation of the LMP1/LMP2 locus of Epstein-Barr Virus by mutation of a single CTCF-Cohesin binding site. J Virol 2014;88:1703–13. 10.1128/JVI.02209-13 24257606 PMC3911611

