# Supplementary figures and images for: Viral loads correlate with upregulation of PD-L1 and worse patient prognosis in Epstein–Barr Virus-associated gastric carcinoma
Source: PLoS One. 2019 Jan 29;14(1):e0211358. doi: 10.1371/journal.pone.0211358 (PMC6350976; doi:10.1371/journal.pone.0211358)

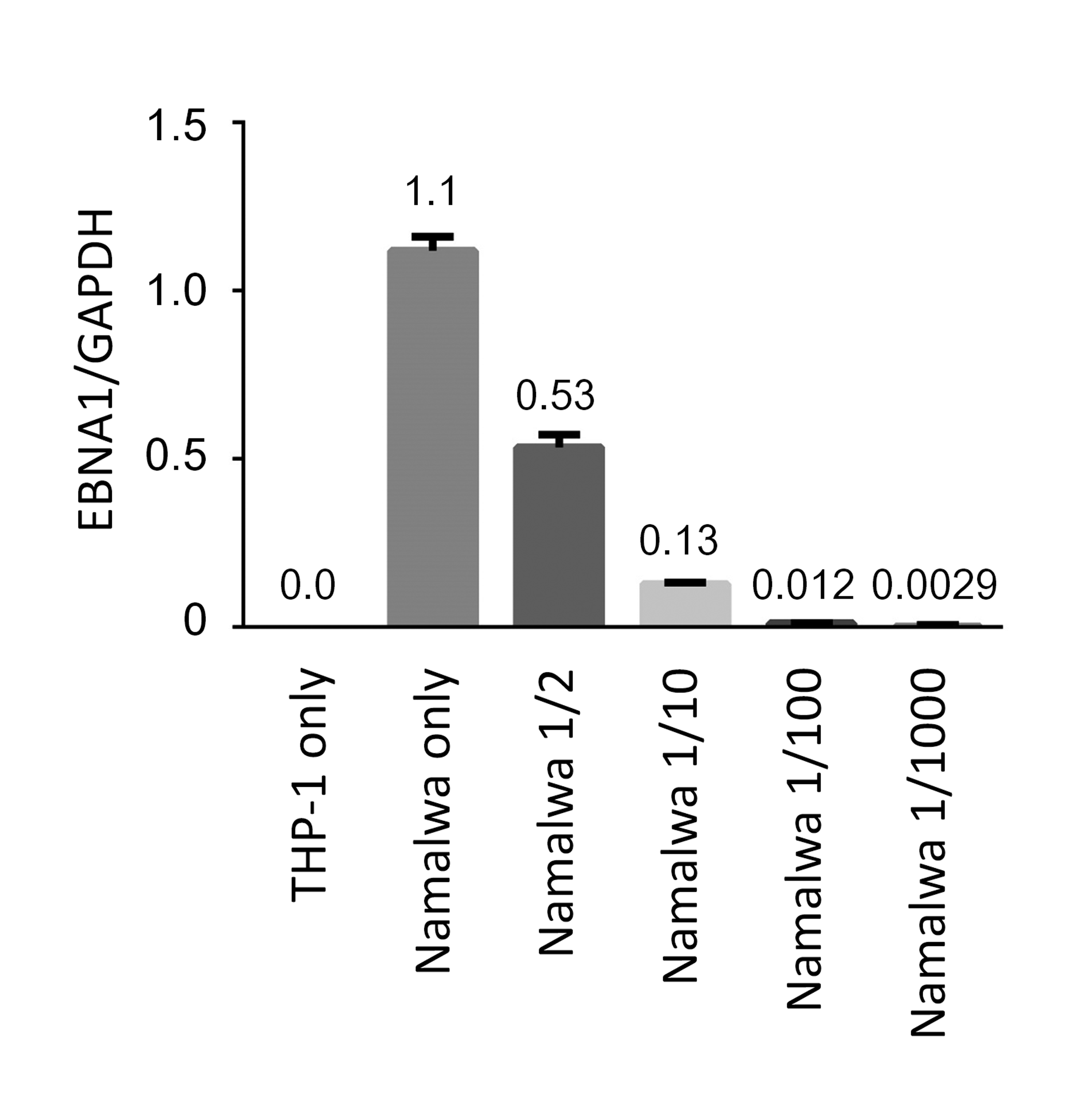

Supplement: S1 Fig — DNA extracted from Namalwa cells (two EBV copies per cell) and THP-1 cells (no EBV infection) was mixed in various proportions (1, 1:2, 1:10, 1:100, and 1:1000), and qPCR was performed. Even in samples with low Namalwa cell DNA concentration (1:1000), we were able to successfully detect EBNA1 DNA. (TIF) [file pone.0211358.s001.tif]

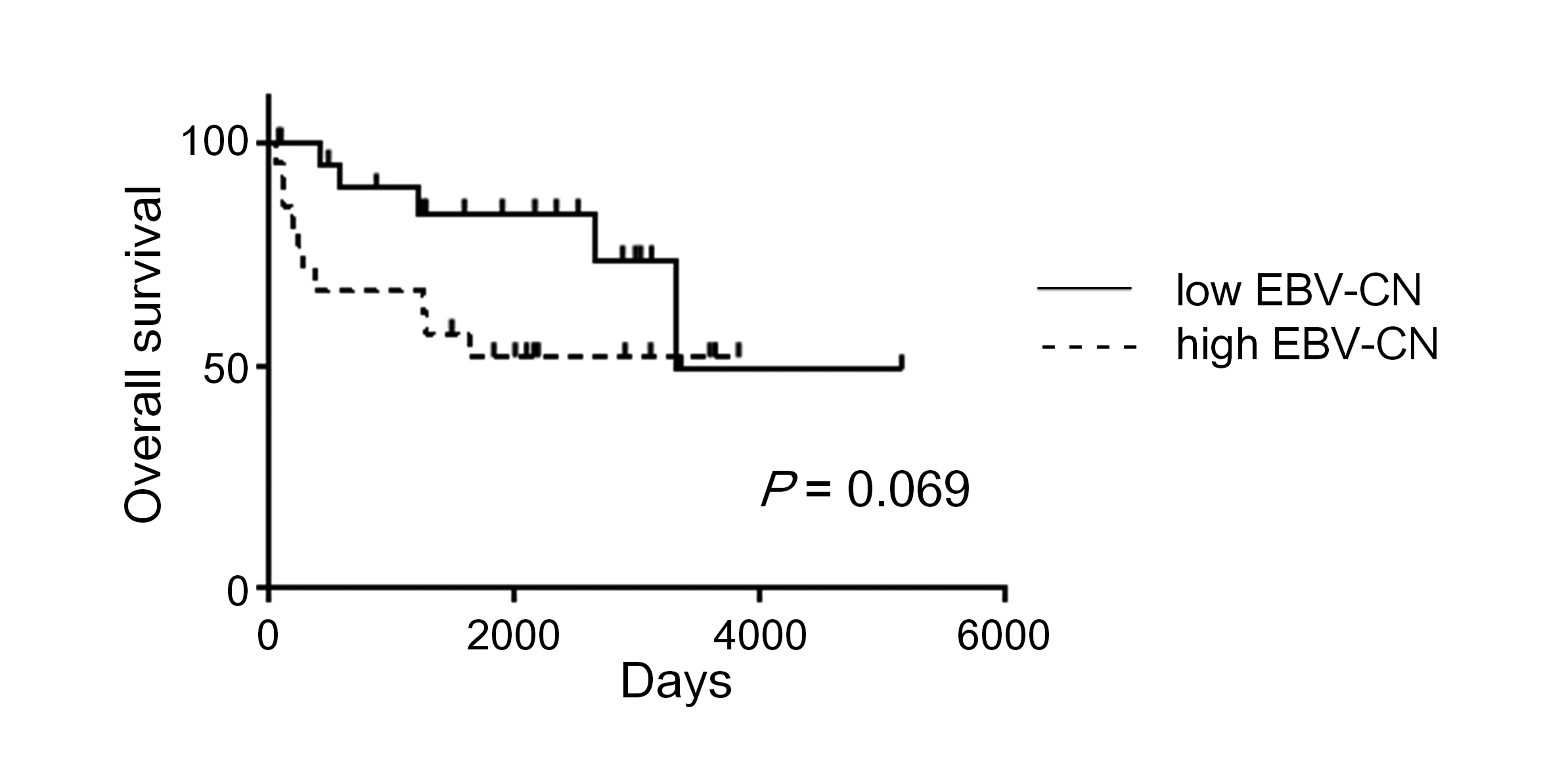

Supplement: S2 Fig — The cases of EBV-associated gastric carcinoma (EBVaGC) were classified into two groups, low and high EBV-copy number per genome (EBV-CN) groups, with the median as a threshold. Overall survival of the patients with EBVaGC was plotted. There was a tendency of worse overall survival in patients with high EBV-CN, although the difference was not statistically significant (P = 0.069). (TIF) [file pone.0211358.s002.tif]
