# Supplementary material for: Viral loads correlate with upregulation of PD-L1 and worse patient prognosis in Epstein–Barr Virus-associated gastric carcinoma
Source: PLoS One. 2019 Jan 29;14(1):e0211358. doi: 10.1371/journal.pone.0211358 (PMC6350976; doi:10.1371/journal.pone.0211358)
Supplement: S1 Table — (DOCX) [file pone.0211358.s003.docx]

**S1 Table. Expression level of viral transcripts in SNU-719 and NCC-24.**

| Cell line | SNU-719 | NCC-24 |
| --- | --- | --- |
| EBV-CN | 42.0 | 1.1 |
| LMP-2A | 1 | 0.33 ± 0.11 |
| EBER1 | 1 | 0.19 ± 0.011 |
| EBNA-1 | 1 | 0.098 ± 0.013 |
| BART-4 | 1 | 0.020 ± 0.0029 |
| BART-7 | 1 | 0.0097 ± 0.00087 |

EBV-CN, EBV copy number per genome.

To examine whether high EBV-CN leads to higher expression level of viral transcripts, we measured expression level of LMP-2A and two viral microRNAs (ebv-mir-BART4-5p and ebv-mir-BART7-3p) in two EBVaGC cell lines (SNU-719 and NCC-24). RNA was extracted from these cell lines with ISOGEN II (Nippon Gene, Toyama, Japan). qPCR of LMP-2A was performed with primers “GCGGGCAGAGGAAGTATGAATC” (forward) and “GTGAAACACGAGGCGGCAATAG” (reverse). qPCR of BART microRNAs was performed with TaqMan miRNA Assays (Applied Biosystems, Foster City, CA, USA).

As a result, expression of LMP-2A, BART4 and BART7 were higher in SNU-719 (high EBV-CN) than NCC-24 (low EBV-CN) as shown in Supplementary Table S1.
